# From adaptation to overload: how climate risks affect public health?—evidence from China

**DOI:** 10.3389/fpubh.2026.1887205

**Published:** 2026-07-24

**Authors:** Shuai Liang, Huanhuan Jiang, Peng Wang, Maomao Zhang

**Affiliations:** 1 School of Applied Economics, Guizhou University of Finance and Economics, Guiyang, China; 2 Western Modernization Research Center, Guizhou University of Finance and Economics, Guiyang, China; 3 College of Public Administration, Huazhong University of Science and Technology, Wuhan, China; 4 College of Urban and Environmental Sciences, Central China Normal University, Wuhan, China

**Keywords:** climate risk, digital financial inclusion, energy structure optimization, nonlinear relationship, public health

## Abstract

Climate risks (CR), particularly extreme climatic events, are systematically threatening global public health (PH). However, most of the existing studies focus on the linear relationship between a single climate factor and health outcomes, lack in-depth investigation of the nonlinear relationship between the two, and pay little attention to the multi-dimensional adjustment of socio-economic factors. This paper collects panel data of 30 provinces in China from 2011 to 2023, uses ArcGIS and the quantile method to visualize the spatial and temporal evolution pattern of provincial CR and PH. By constructing a province-year fixed effect model with a square term and a moderating effect model, the nonlinear relationship between the two is systematically explored, and the moderating mechanism and heterogeneity of different social factors are identified. The results show that the response of PH to CR is first actively adapted and then significantly inhibited, showing a significant inverted U-shaped relationship. Digital inclusive finance (DIF) and residents’ income level (RI) make the inverted U-shaped curve steeper and push the inflection point to the right, showing the characteristics of efficiency-enhanced adjustment; the energy structure optimization (ESO) makes the curve more gentle and the inflection point move to the left, which is manifested as risk-releasing regulation. According to heterogeneity analysis, environmental regulations intensity and green spaces construction have mitigated the health impacts of CR through buffering effects, while green technological innovation has contributed to the manifestation of the inverted U-shaped relationship. This study confirms the existence of an adaptive threshold in the climate-health system resilience, and elucidates three distinct moderating pathways—financial resilience, economic resources, and energy infrastructure—each operating through different mechanisms.

## 1 Introduction

Against the backdrop of the accelerated transformation of the climate system, the frequency, intensity, and duration of extreme weather events are all on the rise, and human health is exposed to unprecedented environmental pressure. In 2024, the global average temperature exceeded the pre-industrial level by 1.55 °C, surpassing the 1.5 °C temperature control target set by the Paris Agreement for the first time. Extreme climate change has a huge impact on health. In China alone, the number of heat-related deaths in 2024 will be 1.7 times the average annual level of the base period (1986–2005). The per capita loss of heat-related labor productivity, safe outdoor exercise time, and sleep will increase by 49.1, 85, and 14.9%, respectively, further intensifying the economic pressure on the country (1). Facing the severe challenge of climate health risks, the international community has moved from consensus to action. In 2025, the 78th World Health Assembly adopted the Global Plan of Action on Climate Change and Health, providing a specific roadmap for countries to incorporate health issues into their national climate commitments and promoting the implementation of policies that view the climate crisis as a health crisis.

In China, climate health governance has been incorporated into the national strategic framework, which explicitly requires the development of meteorological health impact forecast and risk early warning models for the population, and provides refined risk warning services by region, season and population to the general public, medical institutions and older population care institutions. Meanwhile, the urbanization process has brought about economic development and social progress, but it has also been accompanied by a series of PH challenges, such as air pollution, the heat island effect, noise exposure, increased psychological stress, and insufficient physical activity (2–4). In this context, a fundamental scientific question remains unanswered: Does the impact of climate risk (CR) on public health (PH) take on some complex non-linear form? Is this nonlinear relationship regulated by different social factors? This not only concerns the scientific assessment of climate health risks, but also directly determines the proactive response strategies of PH resources under different social factors.

The risks related to climate change are huge, threatening the livelihoods and even life safety of countless people, but some of its economic consequences are still immeasurable at present (5). CR caused by climate change refers to the direct damage to economic and social activities and the human living environment; they are caused by hurricanes, droughts, floods, heat waves and physical events directly related to climate warming (6). Unlike the traditional single perspective on meteorological disasters, modern climatic physical risks cover multiple dimensions such as extreme temperatures, extreme precipitation, drought and wind speed, and have a systemic impact on PH through multiple paths. In China, at the level of high-temperature related risks, for every 1 °C increase in environmental temperature, the risk of injury increases by 1.2%. Among them, men, children aged 0–4, agricultural workers and animal injury categories are particularly sensitive to temperature changes (7). Conversely, sudden drops in temperature and prolonged low temperatures can cause blood vessels to constrict, blood pressure to rise, and heart rate to accelerate, which can easily lead to cardiovascular and cerebrovascular diseases such as myocardial infarction and stroke. Alarmingly, the compounding of extreme weather events exacerbates health risks. For instance, compound diurnal heatwaves and combined hot-dry events are associated with higher mortality risks than isolated extreme climate events (8, 9).

Considerable research has established a solid foundation for exploring how CR relates to PH. Notably, vulnerability in health outcomes tied to climate conditions arises from the complex, multidimensional interaction among social, economic, geographic, and health infrastructure factors (10), with disadvantaged populations—including those living in poverty, outdoor workers, women and children, and communities in ecologically fragile areas—bearing a disproportionate share of the health burden. PH, as a multidimensional construct, encompasses both the foundational healthcare capacity of a society and specific population health outcomes, such as life expectancy at birth, mortality rates, and infectious disease incidence. These dimensions may respond differently to climate physical risk. Methodologically, the distributed lag nonlinear model (DLNM) has proven valuable in capturing the nonlinear characteristics of exposure–response relationships as well as the lagged distribution structure of risks (11). Empirically, the non-accidental mortality risks associated with daytime heatwaves and nighttime heatwaves increase significantly only after cumulative heat exposure reaches relatively high levels (12). These pieces of evidence collectively point to the possibility that there may also be a complex nonlinear correlation between CR and PH.

Nevertheless, there are still areas for improvement in the existing literature. Firstly, there is a lack of aggregated assessment of CR. Most studies focus on a single temperature factor or heat waves caused by high temperatures, while in reality, the superimposition of multiple risks is the norm. The combined nonlinear effects of anomalous climate events on PH remain to be systematically identified. Secondly, existing research has largely adopted a micro-level perspective, reporting average effects on mental health, specific disease incidence, mortality, or self-rated health status through questionnaires, without comprehensively considering the multiple dimensions of PH. Healthcare system capacity and population health outcomes represent two qualitatively distinct health dimensions: the former reflects systemic resilience, whereas the latter captures ultimate health endpoints. However, few studies have examined these dimensions simultaneously within a unified analytical framework. Thirdly, there are limitations regarding the research perspective. Social factors have only been empirically examined in a small subset of climate–health studies and often without systematic treatment. The social gradient in health inequality has become further amplified amid the climate crisis, yet empirical investigations integrating multiple moderating pathways—such as digital finance, economic endowments, and clean energy—remain limited.

To address the aforementioned research limitations, this work adopts a perspective focused on the resilience of the climate-health system, drawing on panel data covering 30 Chinese provinces (Tibet not included) from 2011 to 2023, operationalizing PH along the capacity of the healthcare system and the health status of the population, which reflects the level of resilience of health systems in each region. In tandem, CR are examined from four dimensions: extreme high temperature, extreme low temperature, extreme precipitation and extreme drought. By constructing a fixed-effect model containing square terms, the nonlinear relationship between CR and PH is explored. The reason for choosing this research perspective is that CR has been proven to be a key driving factor for the outbreak of various infectious diseases such as dengue fever, malaria, and intestinal diseases. Moreover, the social medical infrastructure and the health status of residents constitute the prerequisite conditions for the prevention and control of infectious diseases. Furthermore, three moderating variables, namely digital inclusive finance (DIF), residents’ income level (RI), and energy structure optimization (ESO), are introduced to construct a logical chain of “finance—economy—energy” under the framework of social development, in order to identify the realization mechanism of the inverted U-shaped form. Lastly, a multi-dimensional heterogeneity analysis based on environmental regulations, green technological innovation and the green space construction is carried out, aiming to provide theoretical support and empirical evidence for precise climate health adaptation policies. This macroscopic perspective provides empirical evidence for understanding the relationship between CR and PH, with the aim of offering preliminary references for the monitoring and early warning of climate-sensitive infectious diseases as well as the prioritized allocation of health resources.

**Figure 1:**
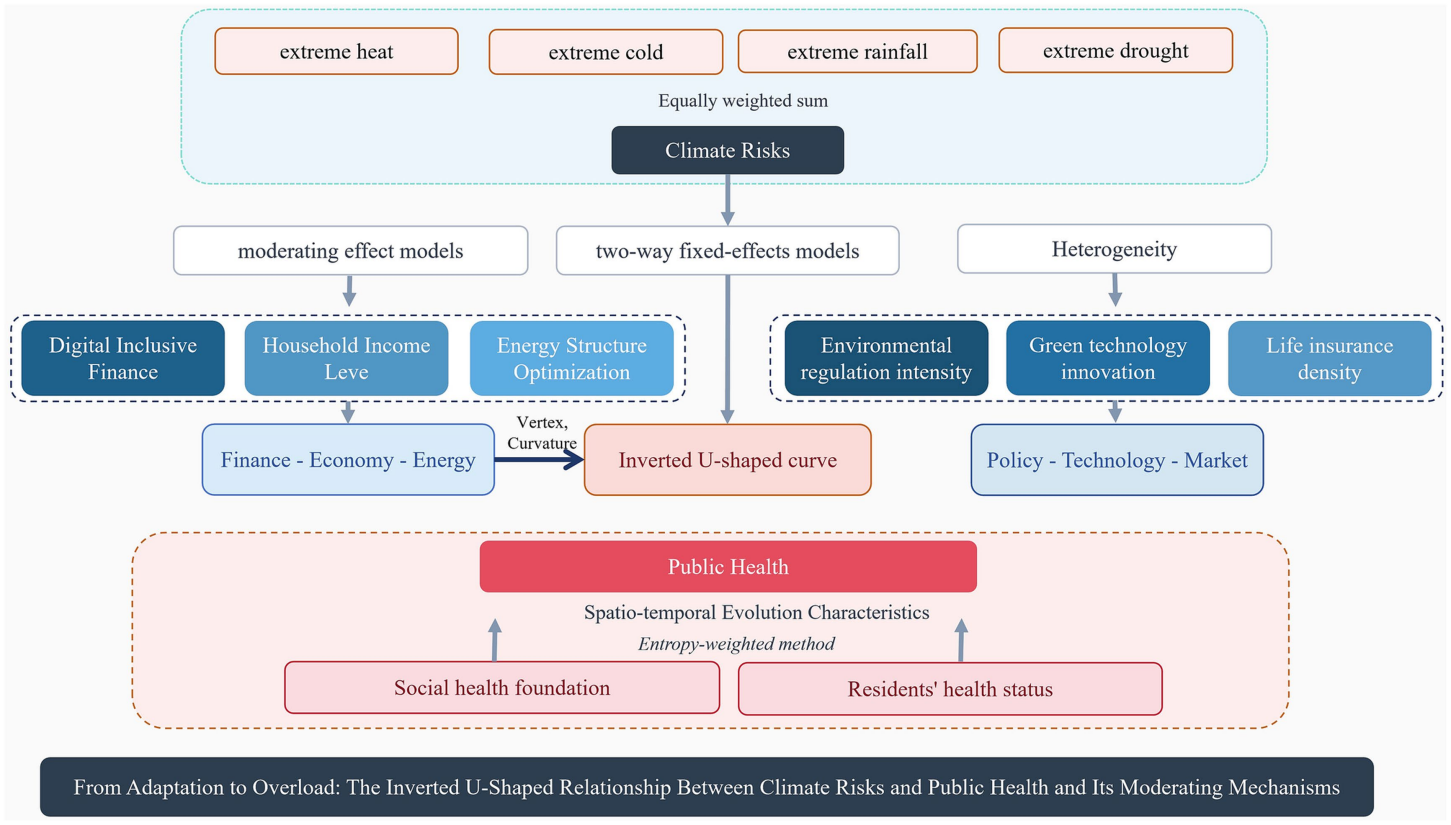
Research design framework diagram.

The framework of the research design is shown in Figure 1.

## 2 Theoretical analysis and research hypotheses

Recent research has gradually revealed that regional differences give rise to distinct patterns of association across areas, and that the relationship between the two is far more complex than previously imagined. Intersecting insights from environmental epidemiology and health system resilience theory suggest that meteorological factors and health outcomes exhibit nonlinear characteristics, rooted in the physiological tolerance limits of the human body, the design capacity of infrastructure, and the emergency response capabilities of social organizations. In summary, future health risks are not merely a function of climate change’s direct impacts. They also hinge on the vulnerability of individuals and communities to these impacts, the readiness of healthcare systems, and the ability to competently manage the risks involved (13).

### 2.1 Climate risk and public health

At the healthcare system level, potential nonlinear associations related to CR have already been identified. For instance, the health damages caused by non-optimal temperatures translate directly into increased healthcare utilization, thereby giving rise to a nonlinear relationship between temperature and medical expenditures at the macro level (14). Specifically, both low and high temperatures drive up healthcare spending, whereas regions with intermediate temperature ranges exhibit relatively lower expenditure levels, displaying a typical U-shaped pattern. Globally, it is now widely recognized that climate change and the associated CR pose major threats to human health, and building greater resilience and adaptive capacity in the face of climate-related hazards yields positive outcomes for human health and well-being (15). Key elements of such efforts encompass sound governance and leadership, heightened public awareness, the strategic distribution of resources, readiness for emergencies, and the provision of robust healthcare services (16). Furthermore, a numerical analysis of the wind load response of skylight systems in healthcare facilities under arid climate conditions illustrates from an engineering perspective that, even under extreme environmental conditions, sound structural design can provide a certain buffer capacity (17). In other words, within the carrying capacity of a system, risk shocks can be effectively absorbed, which is inherently consistent with the logic of nonlinear responses of PH systems to CR.

In daily operations, hospitals usually maintain a certain amount of idle resources (vacant beds, standby personnel) to cope with regular fluctuations. When the climate shock is weak, this reserve is sufficient to absorb the increase in patients, and their health performance is not significantly affected. During and after extreme weather or climate events, a well-developed medical system can prevent possible injuries, diseases and deaths (18). However, the buffering capacity of the medical system against CR is not unlimited. When the influx of patients exceeds the system’s processing capacity, the situation will undergo a qualitative change. Once long-term and persistent CR exceed the adaptability of individuals and the medical system, they will not only promote the emergence and spread of infectious diseases but also affect medical personnel and infrastructure, triggering health emergencies (19).

In the domain of population health outcomes, physiological adaptation mechanisms to temperature, precipitation, and drought vary across individuals and contexts. Heatwaves can alter blood flow distribution, induce dehydration and hemoconcentration, and increase cardiac load (20), whereas cold spells may trigger vasoconstriction, reduce immune defense capacity, and exacerbate cardiopulmonary burden (21). These two distinct physiological response pathways suggest that the relationship between CR and health may exhibit multiple nonlinearities—that is, different thresholds and response curves exist at the hot and cold ends, rather than a symmetric unimodal pattern. Globally, extreme temperatures, particularly with rising wet-bulb temperatures, are associated with a U-shaped pattern in the burden of hypertensive disorders in pregnancy, which first declines to a minimum and then rebounds (22). Regarding heatwave typology, the non-accidental mortality risks of daytime-only and nighttime-only heatwaves increase significantly only after cumulative heatwave indices reach relatively high levels (75th–90th percentiles), whereas compound diurnal-nocturnal heatwaves exhibit a superlinear increase beyond the 25th percentile (12). In Bangkok, Thailand, significant nonlinear associations have been identified between dengue fever incidence and mean temperature, minimum temperature, humidity, rainfall, and wind speed (23). Notably, risk increases under low humidity, decreases under moderate humidity, and rises again under high humidity, indicating that nonlinear relationships exist not only for temperature but also for humidity and wind speed, etc.

Synthesizing the evidence above, the relationship between CR and PH exhibits nonlinear characteristics. Whether concerning healthcare system capacity or population health outcomes, and whether involving temperature or precipitation, PH responds nonlinearly to the occurrence of different extreme climate events. Simple linear modeling may therefore fail to accurately characterize the health effects of CR. Accordingly, this study proposes:

*H1*: A nonlinear, inverted U-shaped association characterizes the link between CR and PH.

### 2.2 Mechanisms through which CR affects PH

#### 2.2.1 The moderating role of digital inclusive finance

In an era of rising climate vulnerability, DIF, as the digital evolution of traditional finance, has contributed significantly to addressing health and nutritional challenges (24), thereby influencing how well residents are able to withstand and respond to climate-related shocks. On the one hand, DIF enhances household financial resilience against CR by lowering access thresholds to financial services and easing credit constraints (25). When extreme weather events occur, affected households can obtain emergency funds for medical expenditures through digital credit and receive risk compensation through digital insurance, thereby buffering the adverse health impacts of CR. Notably, in the Chinese context, DIF has been shown to significantly mitigate the negative effects of climate variability on income growth by improving the circulation efficiency of the financial system and promoting the adoption of new technologies and resource use efficiency (26). Although this finding focuses on income rather than direct health outcomes, income is a well-established social determinant of health. In remote areas, in particular, climate- and weather-related events raise the transmission risk for infectious diseases and aggravate non-communicable illnesses (27), where limited economic capacity can easily trap households in health-related poverty cycles. Improved income stability implies a greater capacity for households to avoid crowding out health investments under risk shocks, offering indirect support for understanding how digital finance may help modulate health outcomes.

On the other hand, DIF may influence the health recovery process following CR shocks by improving the accessibility of healthcare services and the availability of health-related information. Digital financial services—such as online medical payments, teleconsultation appointments, and health insurance purchases—reduce the transaction costs of obtaining healthcare (28, 29), thereby mitigating the disruptive effects of climate events on healthcare utilization. It should be noted, however, that the effect of digital finance may not follow a simple linear pattern. When CR is low, digital finance enhances health levels by improving daily health management, such as mobile health applications and digital preventive reminders. However, the effectiveness of digital finance depends on the coverage of digital infrastructure and users’ digital literacy. Merely expanding the accessibility of financial services would instead exacerbate the existing structural limitations (30). During high-risk climate periods, if these conditions are not met, digital finance’s reliance on energy consumption and the high upfront investment may become a new vulnerable point (31). Moreover, high-digital finance regions are usually economically developed areas, facing structural vulnerabilities such as an aging population and the urban heat island effect. They may fail due to network disruptions in extreme events, leading to more severe health deterioration.

*H2a*: The inverted U-shaped association linking CR to PH is significantly moderated by DIF.

#### 2.2.2 The moderating role of residents’ income level

Income levels, as the core determinant of an individual’s and a family’s ability to allocate resources, influence the health effects of CR through multiple channels. On one side, residents with relatively high incomes support the purchase of air conditioners and the improvement of insulation conditions in their houses. Moreover, the increase in income encourages residents to actively take preventive medical measures, such as accessing quality medical services and purchasing health insurance, which stimulates the demand for medical care (32). These adaptation measures can effectively reduce an individual’s health vulnerability when CR occurs. More direct evidence is that in East China, high income levels effectively reduce the relative risks of cold and heat exposure. For every 10,000 yuan increase in per capita GDP, the risk of heat-related non-accidental death decreases by 0.075% (33). This means that economic resource endowments have changed the health damage transformation of CR by influencing adaptive investment.

On the other side, income levels may affect the resilience of residents and the healthcare system after CR shocks. After experiencing health shocks, high-income groups can mobilize more resources for rehabilitation treatment and long-term health management, thereby reducing the cumulative health damage caused by risk exposure. Conversely, low-income families may face the predicament of medical expenses encroaching on basic living expenses after climate shocks, creating a vicious cycle where health damage and economic pressure reinforce each other. This reflects the differences in individual adaptability resulting from different income levels (34, 35). In low-income and middle-income countries, as income levels increase, residents and the government’s ability to invest in improving housing quality, enhancing medical conditions, and building risk warning systems significantly increases, fundamentally raising the carrying capacity of the health system to cope with climate risks. Relevant studies show that regional income levels are an important driving factor for residents to adopt heatwave adaptation behaviors, and regions with higher incomes typically have stronger climate resilience (36). However, in high-income countries, heat-related mortality rates rise with the increase in a country’s wealth, a finding that breaks the linear thinking that economic development automatically brings health benefits. Although affluent societies have stronger adaptive resources, population aging, the urban heat island effect, and reliance on cooling infrastructure have instead increased the system’s susceptibility to failure in extreme events (37). Given this, there may be a critical point in the moderation of the health effects of CR by income. Before the income level reaches a certain threshold, the increase in income mainly translates into an improvement in adaptability. Beyond this threshold, the lifestyle changes accompanying the increase in income (such as sedentary behavior and air conditioning dependence) may introduce new vulnerabilities. Accordingly, we propose:

*H2b*: RI significantly moderates the relationship between CR and PH.

#### 2.2.3 The moderating role of energy structure optimization

At the aspect of air pollution reduction, the increase in the proportion of clean energy means that the demand for fossil energy such as coal and oil in terminal energy consumption decreases, reducing the emissions of air pollutants such as sulfur dioxide, nitrogen oxides and fine particulate matter (38). These pollutants have been confirmed to be significantly associated with the risk of death from heart and lung diseases and respiratory diseases. Under the goal of carbon neutrality, the replacement of fossil energy with clean energy has achieved sustainability and applicability in energy production and reduced health risks to the population (39). When CR occurs, people with higher health levels have a stronger ability to withstand extreme weather events, which means that energy structure optimization may buffer the marginal health damage of CR by improving basic health conditions. Panel data analysis of developing countries reveals a U-shaped association between clean energy poverty and health vulnerability; the improvement of clean energy accessibility can reduce the sensitivity of health vulnerability to energy poverty (40). Thus, ESO not only improves environmental quality through emission reduction but also enhances climate resilience by reducing the system’s sensitivity to energy fluctuations.

At the level of systemic resilience, the influence of energy structure on climate adaptive capacity becomes salient. Although integrating renewable energy makes energy management more challenging, it is even more critical for ensuring grid stability (41). The distributed nature of renewable energy resources (e.g., rooftop photovoltaics and small-scale wind power), can provide a more resilient electricity supply during extreme weather events (42), thereby supporting the operation of medical facilities and meeting residential cooling demands. In contrast, regions that rely heavily on centralized fossil fuel-based grids may face power outage risks during extreme weather events, which can amplify the health damages of CR. Particularly at higher levels of CR, clean energy systems reduce systemic risk stemming from the failure of a single energy infrastructure component by enhancing the diversity and decentralization of energy supply, thereby helping to avoid a precipitous increase in health damages. Accordingly, this study proposes:

*H2c*: ESO exerts a notable moderating influence on the nonlinear, inverted U-shaped pattern observed between CR and PH.

## 3 Data and methods

### 3.1 Sample and data sources

This research takes the CR and PH in the provincial regions of China as the main objects of investigation. Considering the availability and authenticity of the data, panel data of 30 provinces in China (excluding the administrative regions of Tibet, Hong Kong, Macao and Taiwan) from 2011 to 2023 are selected, and linear interpolation is performed on some missing data. Data on extreme climate events are obtained from the National Oceanic and Atmospheric Administration (NOAA) and China’s Blue Book on Climate Change for the respective years. PH data are primarily drawn from the China Health Statistics Yearbook and the China Health and Wellness Statistics Yearbook. PM2.5 concentration data are derived from the China High-Resolution and High-Quality Surface Air Pollutant (CHAP) dataset. Other variables are mainly sourced from the EPS database and various provincial statistical yearbooks. Administrative boundary data for China are obtained from Tianditu.^1^ To mitigate the influence of heteroscedasticity, robust standard errors are employed in all regression models.

### 3.2 Variable selection

#### 3.2.1 Dependent variable: public health (PH)

PH involves a complex system of health interactions. Beyond simple physical and mental health, it is closely tied to medical care, environmental exposures, social behaviors, and well-being related to other social factors. A single indicator is insufficient to fully capture the multidimensional nature of PH. Drawing on relevant research (43, 44), and taking into account the validity, availability, and representativeness of indicator data, this study constructs a PH evaluation index system from two dimensions: social health infrastructure and population health outcomes (Table 1). This not only takes into account the fundamental supporting conditions necessary for maintaining PH, but also considers the actual improvement of residents’ health, which is conducive to more objectively reflecting the PH level at the macro level.

**Table 1:** The comprehensive evaluation index system of public health.

Primary indicator	Secondary indicator	Indicator description	Attribute
Social health foundation	Medical resources	Number of hospital beds per thousand people	+
		Number of practicing physicians per thousand people	+
	Health services	Number of community health centers per ten thousand people	+
	Financial input	Per capita fiscal expenditure on healthcare	+
	Social security	Enrollment rate in basic medical insurance (%)	+
Residents’ health status	Health outcome	Population survival rate (%)	+
		Infant mortality rate (%)	−
	Expected health	Per capita life expectancy (years)	+
	Health prevention	Population density for health check-ups (people/km2)	+
	Disease burden	Incidence rate of notifiable infectious diseases (per 100,000)	−

The social health foundation reflects the supply capacity and guarantee level of health services in a region, representing the foundational input into PH. PH is not merely the result of individual choices. It also depends on healthcare accessibility and the availability of health services and serves as an important indicator of residents’ quality of life (45, 46). Therefore, the measurement of the social health foundation starts from four areas: medical resources, health services, financial input and social security. Specifically, the number of beds in medical and health institutions per 1,000 people (beds), the number of practicing physicians per 1,000 people (physicians), the number of community health service centers per 10,000 people (centers), the per capita fiscal expenditure on medical and health care (yuan), and the participation rate of basic medical insurance (%) are, respectively, selected for measurement.

The health performance of residents is the actual output of PH and directly reflects the direct results of PH, directly reflecting the end results of PH. Ultimately, promoting positive and healthy preventive behaviors among residents through science, it effectively reduces the burden of various diseases and maximizes health-related “expected outputs” (such as lowering mortality rates or increasing life expectancy). In a word, it is the fundamental value pursuit for effectively improviting PH and ensuring the sustainability of the health system (47–49). To comprehensively characterize population health outcomes, this study examines four aspects: health outcomes, expected health, preventive health, and disease burden. The following proxy indicators are, respectively, employed: population survival rate (%), infant mortality rate (%), per capita life expectancy (years), health examination population density (persons/km^2^), and the reported incidence rate of notifiable Class A and B infectious diseases (per 10,000 population).

After constructing the index system, this paper uses the entropy method to calculate the PH comprehensive index. This method determines the weights based on the degree of dispersion of each index in the sample data, which can avoid the deviation that may be caused by subjective weighting. The specific calculation steps are as follows:

To eliminate the influence of different index units and unify the direction, the indicators are linearly transformed through the range standardization method. Here, the standardization formula for positive indicators is Equation (1):


$$X_{\mathrm{ij}}=(x_{\mathrm{ij}}-x_{\mathrm{ij}}^{\min})/(x_{\mathrm{ij}}^{\max}-x_{\mathrm{ij}}^{\min})$$ (1)

The standardization formula for negative indicators is Equation (2):


$$X_{\mathrm{ij}}=(x_{\mathrm{ij}}^{\max}-x_{\mathrm{ij}})/(x_{\mathrm{ij}}^{\max}-x_{\mathrm{ij}}^{\min})$$ (2)

Where $x_{\mathrm{ij}}$ is the *j*-th indicator of province I, $x_{\mathrm{ij}}^{\max}$ and $x_{\mathrm{ij}}^{\min}$ represent the maximum and minimum values of province I in all years. After standardization, the weights are assigned through the entropy method. First, calculate the proportion of the *j*-th indicator of province *i* (as shown Equation (3)):


$$P_{\mathrm{ij}}=\frac{X_{\mathrm{ij}}}{\sum_{i=1}^{n}X_{\mathrm{IJ}}}$$ (3)

Where n is the total number of sample provinces. Secondly, calculate the information entropy of the *j*-th indicator (as shown Equation (4)):


$$e_{j}=-\frac{1}{\ln(n)}\sum_{i=1}^{n}[P_{\mathrm{IJ}}\ln(P_{\mathrm{ij}})]$$ (4)

If $P_{\mathrm{ij}}$ = 0, then define Pij ln ( $P_{\mathrm{ij}}$ ) = 0. Then, calculate the information entropy redundancy (difference coefficient) dj = 1 – ej = 1 – ej, and the weight of the *j*-th indicator is Equation (5):


$$w_{\mathrm{ij}}=\frac{d_{\mathrm{ij}}}{\sum_{i=1}^{m}d_{\mathrm{IJ}}}$$ (5)

Where m is the total number of indicators. Finally, calculate the comprehensive public health index of each province in each year through the weighted summation method (as shown Equation (6)):


$$PH_{\mathrm{ij}}=\sum_{j=1}^{m}w_{\mathrm{ij}}\times X_{\mathrm{ijt}}$$ (6)

$PH_{\mathrm{ij}}$ is the comprehensive public health index, with a value ranging from [0, 1], and the higher the value, the higher the PH level.

#### 3.2.2 Independent variable: climate risk (CR)

To measure CR, this study adopts the climate physical risks index developed by Rising et al. (5). Four component indicators are used: days of extreme heat, days of extreme cold, days of extreme rainfall, and days of extreme drought. (1) Taking the period from January 1, 1973 to December 31, 1992 as the base period, the upper and lower 10% percentiles of the historical daily average temperature of meteorological stations were, respectively, taken as the thresholds for extreme high temperature and extreme low temperature, the 95% percentile of rainfall was taken as the threshold for extreme rainfall, and the 5% percentile of daily humidity was taken as the threshold for extreme drought. (2) Based on the above thresholds, calculate the number of days in each province and each year that exceed these thresholds. Then, sum up the number of days for each station within the same province, and divide by the total number of meteorological stations in that province to obtain the arithmetic mean of the four types of extreme climate events. Furthermore, the overall indices of various extreme climates were obtained by using the minimum-maximum standardization method: days of extreme heat (HTD), days of extreme cold (LTD), days of extreme rainfall (ERD), and days of extreme drought (EED). Take extreme hot weather as an example (as shown Equation (7)):


$$\mathrm{HT}D_{\mathrm{it}}=\frac{{\bar{\mathrm{HTD}}}_{\mathrm{it}}-\min_{K=1,k,L=1,l}\{{\bar{\mathrm{HTD}}}_{\mathrm{kl}}\}}{\begin{array}{l} \max_{K=1,k,\;L=1,l}\{{\bar{\mathrm{HTD}}}_{\mathrm{kl}}\}\\ -\min_{K=1,k,\;L=1,l}\{{\bar{\mathrm{HTD}}}_{\mathrm{kl}}\} \end{array}}$$ (7)

Here, ${\bar{\mathrm{HTD}}}_{\mathrm{it}}$ represents the arithmetic mean of extreme high temperatures in province i over the t-year period, while K and L, respectively, denote the total number of provinces and the total number of years.

(3) Finally, the four types of indicators were, respectively, weighted by 0.25 and summed up to obtain the climate physical risks index. This index characterizes the diversity of extreme CR from various dimensions. Against the backdrop of China’s vast territory and diverse climates, it not only reflects the risk levels faced by each province but also enables horizontal comparisons across the country.

#### 3.2.3 Moderating variables

##### 3.2.3.1 Digital inclusive finance (DIF)

This article refers to the Digital Inclusive Finance Index (2011–2023) compiled by Peking University for measurement, which covers multiple financial service areas ranging from payment and credit to investment and money funds, comprehensively demonstrating the breadth and depth of financial services at various levels as well as their performance in different regions.

##### 3.2.3.2 Residents’ income level (RI)

RI is measured in the logarithmic form of the per capita disposable income of urban residents. This indicator can reflect the income base available for final consumption expenditure and the degree of living affluence of local residents, and is an important manifestation of residents’ purchasing power, consumption capacity and quality of life (50).

##### 3.2.3.3 Energy structure optimization (ESO)

To comprehensively promote energy conservation and emission reduction, accelerating ESO can help reduce reliance on traditional fossil fuels, increase the proportion of clean energy, lower greenhouse gas emissions and alleviate environmental pollution. It is an important way to achieve the “dual carbon” goals. As the demand for electricity increases, people have started to seek more clean and sustainable energy sources. The growing demand for electricity consumption means that the main force of energy consumption is shifting from coal to electricity. Increasing electrification may be an important way to adjust the energy structure (51). Clean energy includes hydropower, wind power, solar energy, natural gas and nuclear energy. The increase in the proportion of clean energy generation reflects the development status of clean energy and the green and low-carbon degree of the energy structure in a region (52). Therefore, this article measures by the ratio of regional clean energy power generation to total power generation.

#### 3.2.4 Control variables

To alleviate the bias of omitted variables, in this paper, referring to existing literature (53), the following external factors that may affect more PH were controlled to improve the accuracy and validity of the model and results: (1) Transportation infrastructure, represented by the mileage of highways (taking the logarithm); (2) The level of urbanization is characterized by the proportion of the urban population in the region at the end of the year. (3) The upgrading of industrial structure is characterized by the ratio of the output value of the tertiary industry to that of the secondary industry in the region. (4) Travel activity is measured by the ratio of total passenger volume to total population. (5) The level of human capital is measured by the proportion of students currently enrolled in colleges and universities to the permanent resident population at the end of the year. (6) Aging, which is represented by the dependency ratio of the people over 65 (population sample survey).

#### 3.2.5 Descriptive statistics

All variable definitions and their descriptive statistics are shown in Table 2. The average VIF of the variables is much less than 10, and there is no severe multicollinearity. The CR index is concentrated in the range of 0.255 to 0.435, and the PH index is concentrated in the range of 0.132 to 0.253. Although there have been improvements in recent years, the PH level in more than half of the provinces is still relatively low. During the study period, PH and the degree of CR exposure in each province changed significantly.

**Table 2:** Descriptive statistics of variables.

Variable type	Variable	Sample size	mean	Standard deviation	Minimum	25th percentile	median	75th percentile	Maximum
Dependent variable	Public health (PH)	390	0.208	0.103	0.054	0.132	0.186	0.253	0.645
Independent variable	Climate risks (CR)	390	0.347	0.141	0.016	0.255	0.346	0.435	0.748
Moderating variables	Digital inclusive finance (DIF)	390	0.000	0.111	−0.237	−0.082	0.012	0.089	0.218
	Residents’ income level (RI)	390	0.000	0.350	−0.745	−0.253	0.019	0.217	0.994
	Energy structure optimization (ESO)	390	−0.000	0.236	−0.275	−0.175	−0.068	−0.068	0.641
Control variables	Transportation infrastructure	390	11.711	0.855	9.400	11.589	11.975	12.268	12.944
	Urbanization level	390	0.606	0.120	0.350	0.523	0.594	0.665	0.896
	Industrial structure upgrading	390	1.376	0.761	0.527	0.998	1.216	1.426	5.690
	Travel activity	390	0.102	0.060	0.023	0.058	0.083	0.129	0.297
	Human capital level	390	0.021	0.006	0.008	0.017	0.021	0.025	0.044
	Aging	390	16.110	4.793	7.440	12.290	14.945	14.945	30.600

To avoid multicollinearity among variables and to render the coefficients of key core variables more intuitively interpretable, the moderating variables—DIF, RI, and ESO—were centered (i.e., mean-deviated) in this study.

### 3.3 Model specification

Based on the theoretical discussion, a baseline fixed-effects regression model with a quadratic term is employed to account for the nonlinear impact of CR on PH (as shown Equation (8)):


$$\begin{array}{l} {\mathrm{PH}}_{\mathrm{it}}=\alpha_{0}+\alpha_{1}CR_{\mathrm{it}}+\alpha_{2}CR_{\mathrm{it}}^{2}\\ +\alpha_{j}\text{contron}l_{\mathrm{it}}+u_{i}+v_{t}+\varepsilon_{\mathrm{it}} \end{array}$$ (8)

In the equation, i and t denote province and year, respectively; PH_it is the dependent variable representing the level of PH; CR_it is the independent variable, namely the climate risk index; control represents the set of control variables; u_i and v_t denote province and year fixed effects, respectively; and ε_it is the random error term.

To further capture the mechanism underlying the nonlinear relationship (i.e., the inverted U-shape) posited in the theoretical analysis and hypotheses, and to examine whether moderating variables affect the curvature and vertex of the nonlinear curve, the following moderating effect model is specified (as shown Equations (9)-(11)):


$$\begin{array}{l} {\mathrm{PH}}_{\mathrm{it}}=\beta_{0}+\beta_{1}CR_{\mathrm{it}}+\beta_{2}CR_{\mathrm{it}}^{2}+\beta_{3}CR_{\mathrm{it}}\times {\mathrm{DIF}}_{\mathrm{it}}+\beta_{4}CR_{\mathrm{it}}^{2}\\ \times {\mathrm{DIF}}_{\mathrm{it}}+\beta_{5}{\mathrm{DIF}}_{\mathrm{it}}+\beta_{j}\text{contron}l_{\mathrm{it}}+u_{i}+v_{t}+\varepsilon_{\mathrm{it}} \end{array}$$ (9)

$$\begin{array}{l} {\mathrm{PH}}_{\mathrm{it}}=\beta_{0}+\beta_{1}CR_{\mathrm{it}}+\beta_{2}CR_{\mathrm{it}}^{2}+\beta_{3}CR_{\mathrm{it}}\times {\mathrm{RI}}_{\mathrm{it}}+\beta_{4}CR_{\mathrm{it}}^{2}\\ \times {\mathrm{RI}}_{\mathrm{it}}+\beta_{5}{\mathrm{RI}}_{\mathrm{it}}+\beta_{j}\text{contron}l_{\mathrm{it}}+u_{i}+v_{t}+\varepsilon_{\mathrm{it}} \end{array}$$ (10)

$$\begin{array}{l} {\mathrm{PH}}_{\mathrm{it}}=\beta_{0}+\beta_{1}CR_{\mathrm{it}}+\beta_{2}CR_{\mathrm{it}}^{2}+\beta_{3}CR_{\mathrm{it}}\times {\mathrm{ESO}}_{\mathrm{it}}+\beta_{4}CR_{\mathrm{it}}^{2}\\ \times {\mathrm{ESO}}_{\mathrm{it}}+\beta_{5}{\mathrm{ESO}}_{\mathrm{it}}+\beta_{j}\text{contron}l_{\mathrm{it}}+u_{i}+v_{t}+\varepsilon_{\mathrm{it}} \end{array}$$ (11)

Here, the moderating variables influencing the inverted U-shaped relationship are DIF_it, RI_it, and ESO_it, representing DIF, household income level, and energy structure optimization, respectively. All other symbols retain the same interpretations as those defined in Equation 1. Whether a moderating variable significantly affects the vertex of the curve depends on whether *β*₁β₄ − β₂β₃ is significantly different from zero. If β₁β₄ − β₂β₃ > 0, the x-coordinate of the vertex increases as the moderating variable increases; otherwise, it moves to the left. Furthermore, the significance of the moderator’s effect on curvature is determined by β₄, the coefficient on the interaction between the CR quadratic term and the moderator. A significantly negative β₄ (β₄ < 0) indicates steeper curvature and thus a stronger nonlinear CR–PH relationship; a significantly positive β₄ (β₄ > 0) indicates flatter curvature and a weakened inverted U-shaped association.

## 4 Empirical results

### 4.1 Characteristics of changes in CR and PH levels

#### 4.1.1 Spatiotemporal evolution characteristics of CR

**Figure 2:**
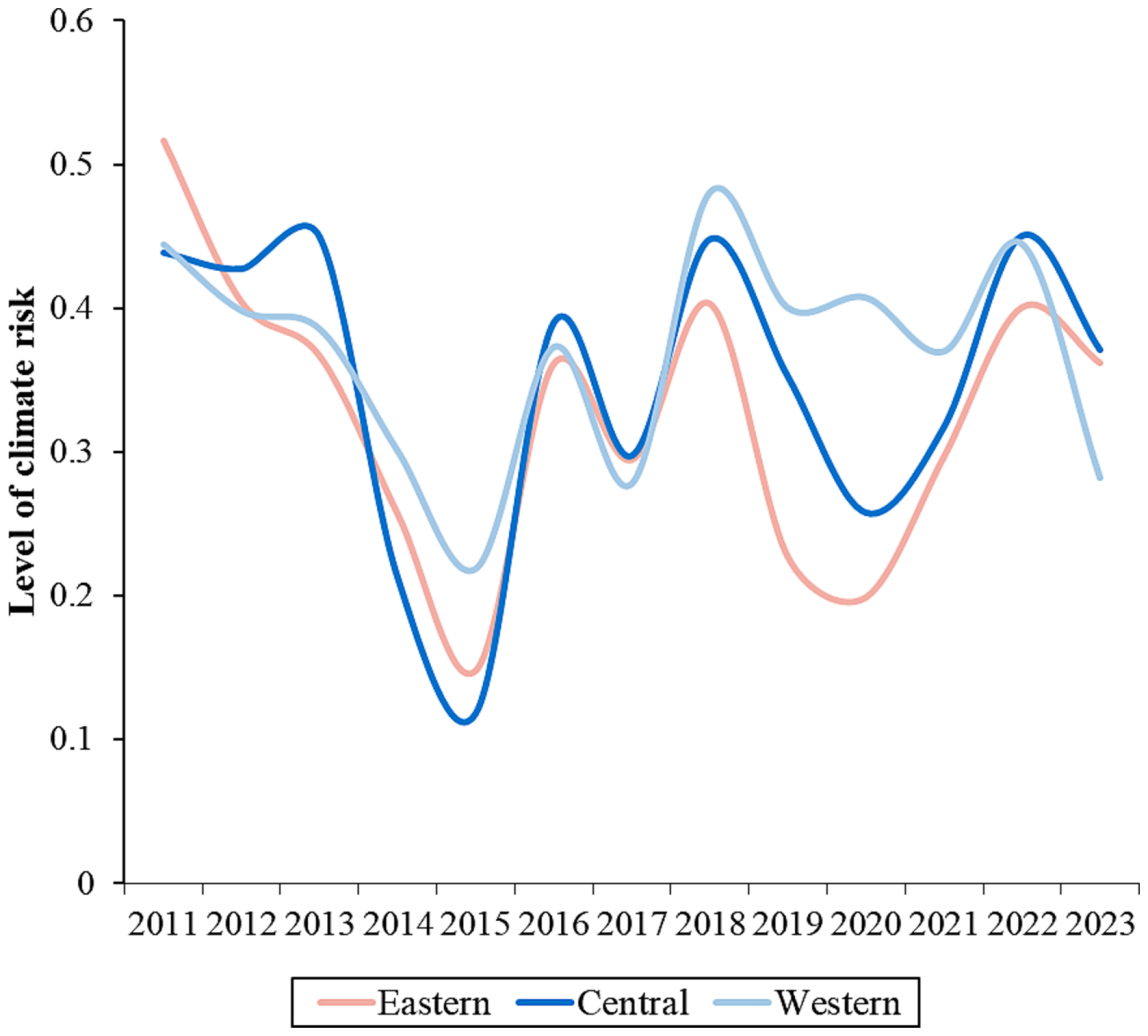
Trends in CR levels in Eastern, Central, and Western China, 2011–2023.

As depicted in Figure 2, due to the uncertainty of climate change, the CR in the eastern, central and western regions fluctuate greatly, presenting a distinct wave-like fluctuation pattern, and the changing trends in the three regions are highly synchronized. This extremely unstable fluctuation requires the health system to be able to quickly mobilize emergency resources during high-risk periods and orderly recover and accumulate reserves during risk decline periods, which puts forward higher requirements for the dynamic adaptability of the PH system.

**Figure 3:**
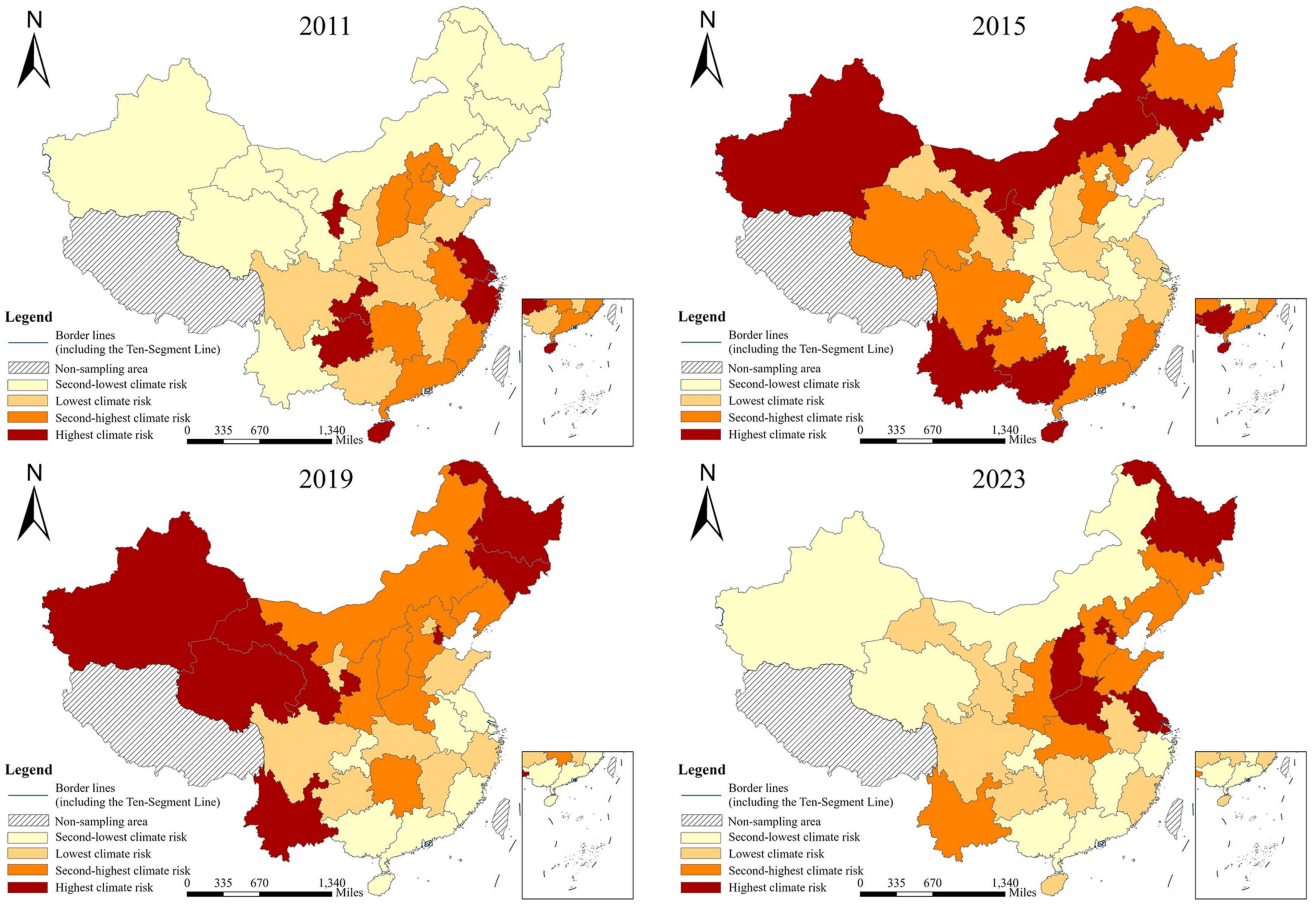
Spatio-temporal evolution of CR across Chinese Provinces, 2011–2023. Base maps from Tianditu (www.tianditu.gov.cn), National Platform for Common GeoSpatial Information Services.

In addition, to more intuitively present the spatio-temporal evolution characteristics of risk climate in various provinces of China from 2011 to 2023, this study utilized ArcGIS 10.8 software to classify climate risk levels into four grades—lowest, second-lowest, second-highest, and highest risk—based on the 25, 50, and 75% quantiles of each year. This is in order to observe and analyze the spatio-temporal evolution characteristics of CR in different regions more intuitively. As described in Figure 3, between 2011 and 2023, no clear regular pattern emerged in the spatiotemporal distribution of CR across China. Higher and above CR levels first occurred predominantly in the eastern coastal regions, then shifted to the southwestern and northern regions. In 2019, risks further concentrated in the north, subsequently moving toward the northeast.

#### 4.1.2 Spatiotemporal evolution characteristics of PH

**Figure 4:**
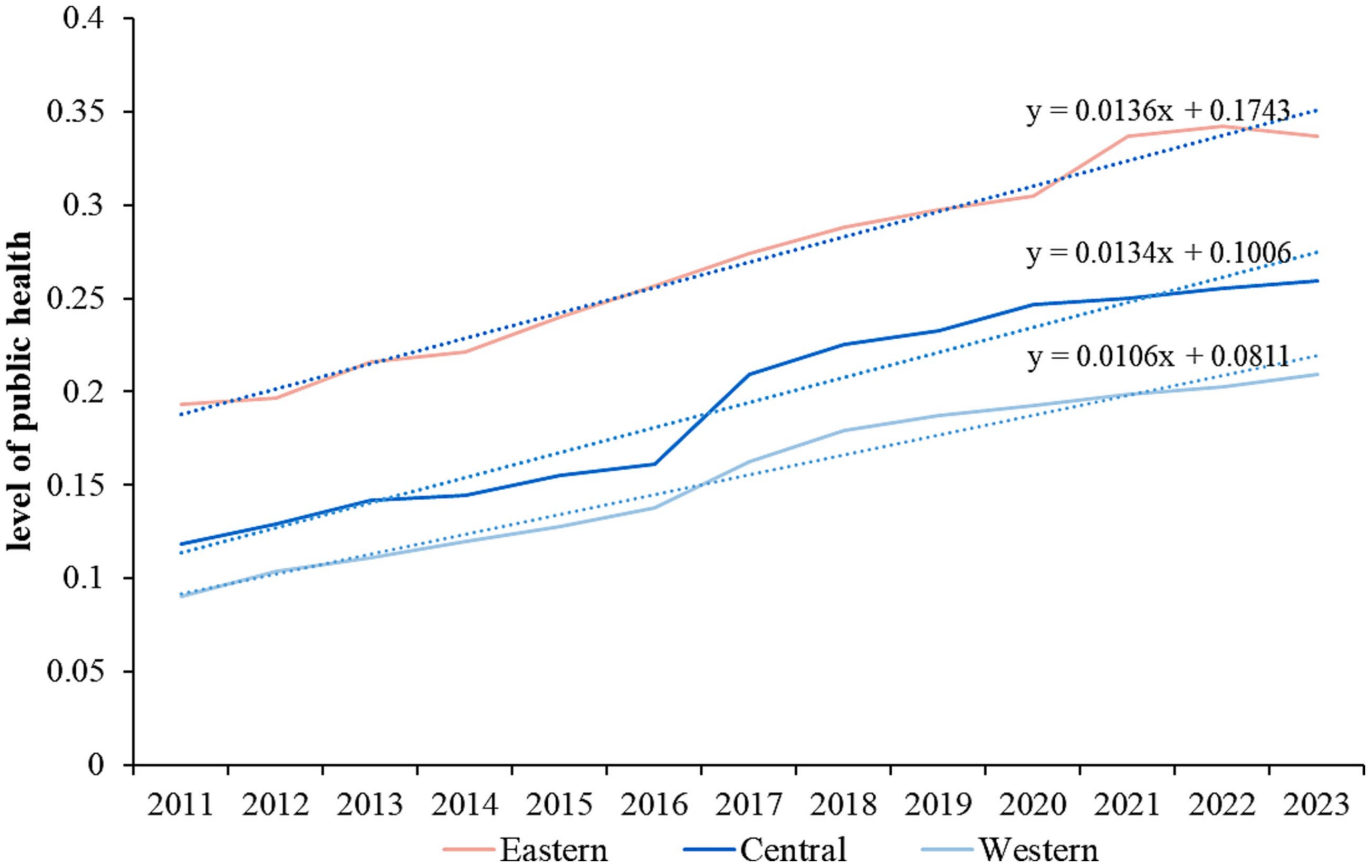
Trends in PH levels in Eastern, Central, and Western China, 2011–2023.

As shown in Figure 4, during the study period, PH levels across all regions of China exhibited a continuous upward trend, following a gradient pattern of eastern > central > western regions, with significant regional disparities in PH levels and similar rates of change over time. Given that these regions faced similar trajectories of CR, this observation suggests that the eastern region possesses significantly stronger adaptive capacity and systemic resilience to CR compared to the central and western regions.

**Figure 5:**
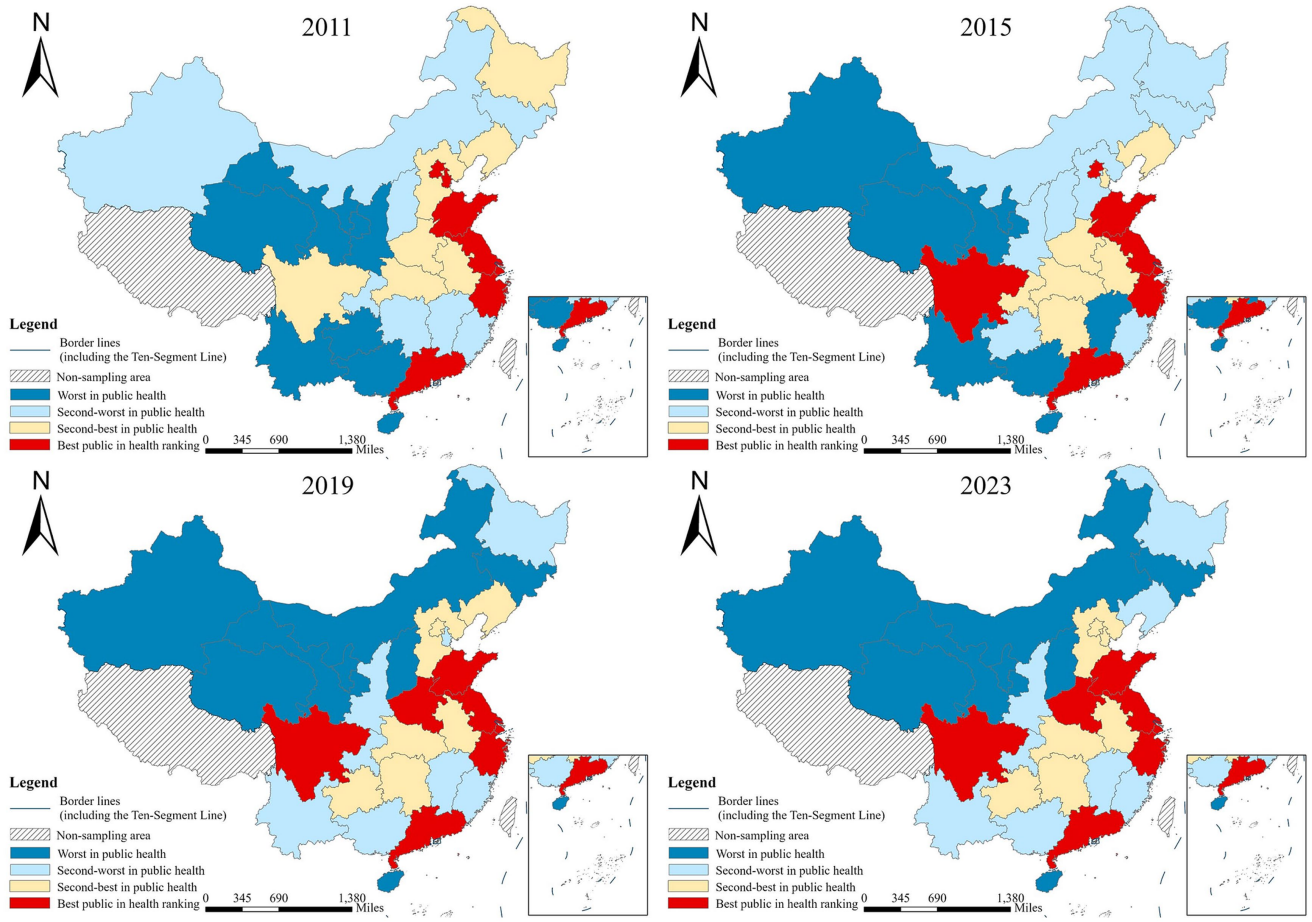
Spatio-temporal evolution of PH across Chinese Provinces, 2011–2023. Base maps from Tianditu (www.tianditu.gov.cn), National Platform for Common GeoSpatial Information Services.

Following a similar approach, PH is classified into four categories: poorest health, poorer health, good health, and excellent health. As reported in Figure 5, during the period from 2011 to 2023, the spatial pattern of China’s PH level presented a highly stable feature: the east was better, followed by the central region, and the west was relatively weak, which was consistent with the previous results. Compared with the spatial evolution of CR, PH landscape shows a stronger dependence on social development, which is highly consistent with the spatial distribution of China’s economic development level and medical resource allocation.

### 4.2 Baseline regression results

Guided by the F-test and Hausman test results, it is determined that the bidirectional fixed-effect model should be given priority in this paper. To verify the nonlinear relationship between the two control variables, CR and its quadratic terms were successively incorporated into the model. It should be noted that the number of cross-sectional samples in this study is 30 provinces. After testing, there is no cross-sectional dependence (*p* > 0.1). When the number of clusters is small (*N* < 50), the clustered robust standard errors may have a downward bias (54). Therefore, this paper mainly adopts heteroscedastic robust standard errors and also reports the robust standard errors for the province clustering.

As shown in Table 3, the significance levels of the core coefficients under the two standard errors are basically the same, indicating that the conclusions of this paper are not sensitive to the choice of standard errors. As shown in column (3), the coefficient of the primary term of CR is 0.089, and the coefficient of the quadratic term is −0.149, both of which are significant at least at the 5% level, preliminarily indicating an inverted U-shaped relationship between them. In addition, according to the utest inverted U-shaped relationship formal test (55), (1) *α*2 was significantly less than 0, (2) the slope on the left side of the corresponding fitting curve was significantly positive, and the slope on the right side was significantly negative. Overall, there was an inverted U-shaped trend (*p* = 0.006). (3) The horizontal coordinate of the vertex (−α 1/2α 2) is 0.298, which is within the upper and lower bound intervals of CR [0.016, 0.748], and the corresponding 95% confidence interval is also within the upper and lower bound intervals of it. These results further formally verify the significant inverted U-shaped trend between CR and PH, thus supporting Hypothesis 1.

**Table 3:** Benchmark regression results.

Variables		(1)	(2)	(3)
		Heteroscedasticity robust standard error	Provincial clustering standard error	Heteroscedasticity robust standard error
CR			0.089** (0.043)	0.089** (0.035)
C*R*^2^			−0.149** (0.055)	−0.149*** (0.044)
Transportation infrastructure		−0.093*** (0.020)	−0.102** (0.044)	−0.102*** (0.021)
Urbanization level		0.324*** (0.122)	0.324 (0.258)	0.324*** (0.120)
Industrial structure upgrading		0.008 (0.010)	0.007 (0.021)	0.007 (0.010)
Travel activity		0.741*** (0.127)	0.761*** (0.230)	0.761*** (0.115)
Human capital level		0.540 (0.928)	0.774 (2.130)	0.774 (0.915)
Aging		0.004*** (0.001)	0.003** (0.001)	0.003*** (0.001)
Constant		0.946*** (0.220)	1.043** (0.464)	1.043*** (0.230)
Inverted U-shaped nonlinearity test		0.089**	0.089**	0.285
	Vertex		0.298	0.298
	Upper limit of the 95% confidence interval		[0.199, 0.397]	[0.220, 0.376]
	Slope at the left endpoint		0.084***	0.084***
	Slope at the right endpoint		−0.135***	−0.135***
Observations		390	390	390
Within *R*^2^		0.245	0.278	0.278
Province-year fixed		yes	yes	yes

Robust standard errors in parentheses; ***, **, and * indicate that the results are significant at the 1%, 5%, and 10% levels, respectively.

**Figure 6:**
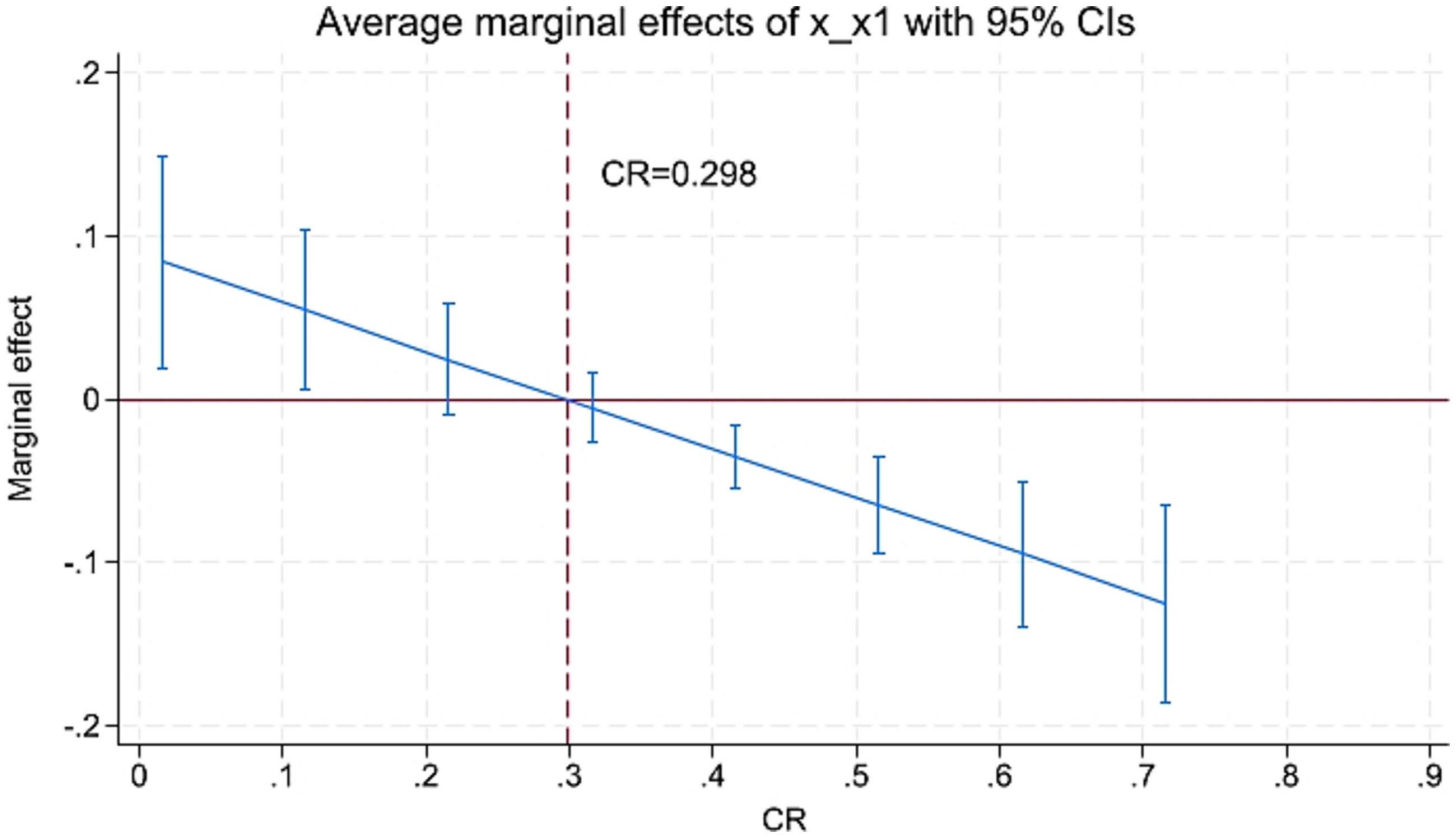
The marginal effect of CR on PH.

**Figure 7:**
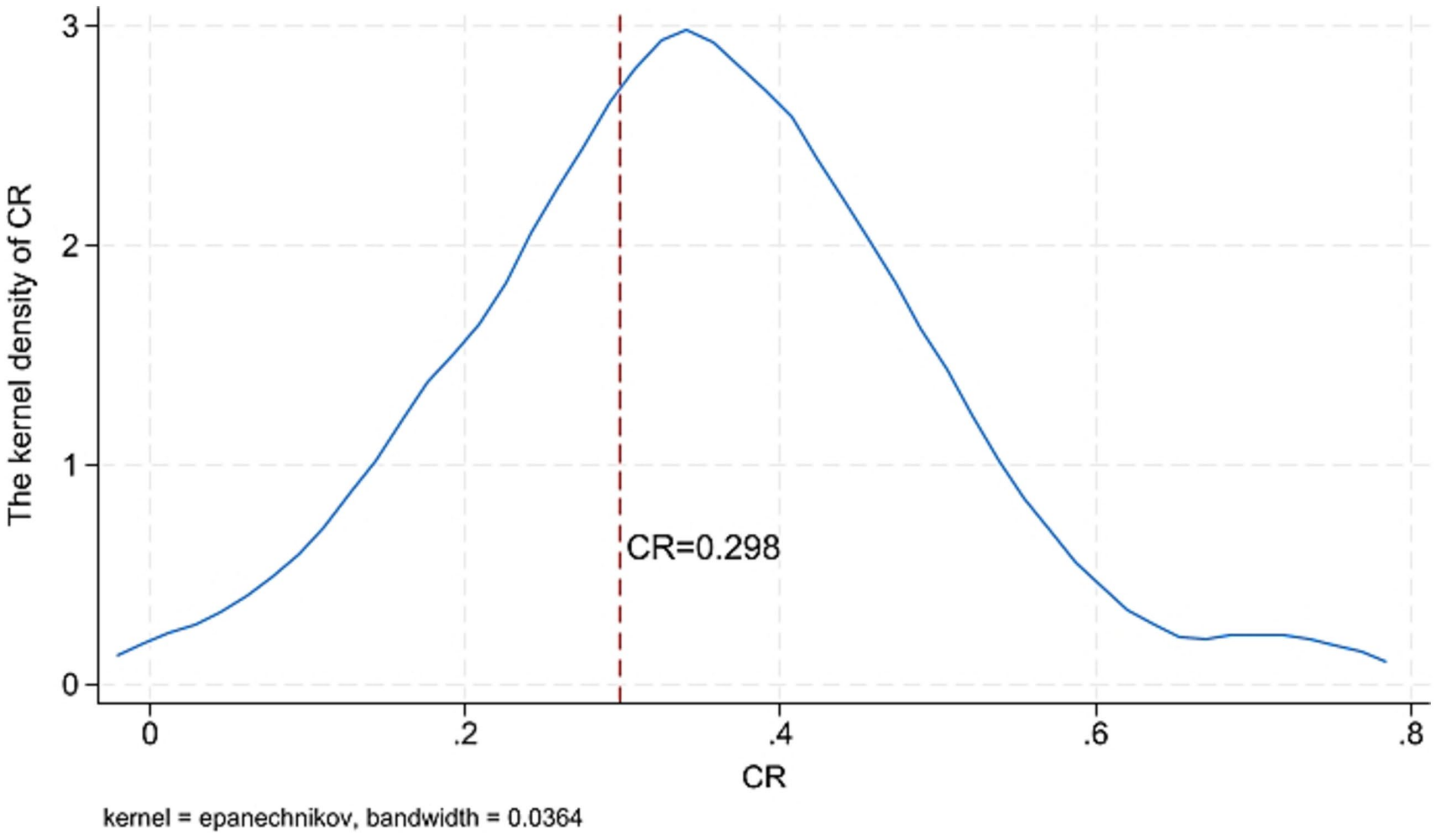
The kernel density distribution of CR.

To further verify this nonlinear relationship between CR and PH, this paper calculated the marginal effect of CR on PH and verified the climate risk distribution around the extreme points. Among them, the marginal effect graph within the 95% confidence interval (see Figure 6) indicates that when CR rises from a low level, the marginal effect is positive but gradually weakens; when it reaches the peak and further strengthens, the marginal effect is 0, then it becomes negative and gradually increases. Moreover, this extreme point is located in an area with high data density. The kernel density plot (Figure 7) shows that the critical point precisely falls in the peak area of the density curve, and the corresponding kernel density value is approximately 2.60–2.80. Further quantitative tests also indicate that the proportion of samples within the neighborhood ±0.05 of the critical point reaches 27.18%. These results strengthen the judgment that there is an inverted U-shaped relationship between CR and PH.

At the theoretical level, this finding supports the adaptive capacity threshold hypothesis. Additionally, this is highly consistent with the thermal effect found by Wang et al. (56) in a study of 122 communities in China, which presents an “inverted U-shaped with a right tail” pattern. When the CR level is relatively low, it may exert an impact on PH through the following several ways: Firstly, the government response, such as government departments initiating climate health warning mechanisms and increasing public health investment; secondly, the medical resource allocation, medical institutions adjusting resource distribution based on risk levels and prioritizing the protection of vulnerable groups; thirdly, the public awareness enhancement, mainly through media publicity or conducting emergency escape drills to prompt residents to enhance health protection awareness; fourthly, the behavioral adaptation, such as residents actively reducing outdoor activities during high-temperature periods and improving ventilation and cooling conditions of their living environments. However, this buffering effect may be limited. When climate risks continue to rise and exceed a certain critical threshold, existing response resources may become strained, infrastructure may face greater operational pressure, and the health benefits of fiscal investment may also decline. At this point, the damage effects brought about by climate risks may gradually accumulate and take the dominant position. This explanation is in line with the logical core of the toxic excitation effect and the stress-performance theory (Yerkes-Dodson’s Law), which states that moderate stress triggers optimal performance, while excessive stress leads to functional decline. We think it is the embodiment of this theory in the field of PH systems responding to CR shocks.

### 4.3 Robustness checks

To enhance the feasibility of the benchmark regression results, the article adopts the following re-regression approach:

(1) Time trends by provinces: To control for the unique time trends specific to each province, we included interaction terms between provinces and time trends in the model to eliminate potential interference from the varying development trajectories of different provinces on the estimation results. (2) Excluding the impact of the epidemic. Given the profound impact of the COVID-19 pandemic on the health system, personnel mobility, the infectious disease reporting mechanism, and fiscal expenditures, this study re-ran the regression after excluding the influence of the sample period from 2020 to 2022. (3) Entropy-based Weighting CR. To test whether the core conclusion is dependent on a specific weighting method for indicators, this paper uses the entropy method to recalculate the CR index for a robustness test, in order to indicate that the conclusion of this paper is not dependent on the specific measurement method of CR. (4) Winsorization. Tail-reduction treatment. Winsorize all variables at levels of 1 and 99% to avoid the possible interference and influence of outliers on the empirical results. (5) Lag period: The health benefits of climate risk may have a time lag. This article selects the first-order lag term regression of climate risk. The detailed results are displayed in Table 4. After considering the interference of the above-mentioned issues, the inverted U-shaped impact of climate risk on PH remains significant, ensuring the robustness of the benchmark regression results.

**Table 4:** Robustness test.

Variables	(1)	(2)	(3)	(4)	(5)
	Time trends by provinces	Excluding the impact of the epidemic	Entropy-based Weighting CR	Winsorization (1.99)	Lagged ter
CR	0.084** (0.035)	0.061** (0.029)	0.163** (0.066)	0.072** (0.028)	
C*R*^2^	−0.141*** (0.044)	−0.097** (0.040)	−0.288*** (0.088)	−0.134*** (0.037)	
L. CR					0.082*** (0.027)
L. C*R*^2^					−0.122*** (0.037)
Control variables	Yes	Yes	Yes	Yes	Yes
Constant	0.919*** (0.234)	0.807*** (0.300)	1.009*** (0.227)	1.013*** (0.220)	0.712*** (0.227)
Observations	390	300	390	390	360
Province-year fixed	Yes	Yes	Yes	Yes	Yes
Within *R*^2^	0.322	0.399	0.276	0.338	0.854

Robust standard errors in parentheses; ***, **, and * indicate that the results are significant at the 1%, 5%, and 10% levels, respectively.

(6) Sensitivity analysis for omitted variables. This work adopts the sensitivity analysis method proposed by Cinelli et al. (57) to assess the potential interference caused by omitted variables. This method calculates the robust value (RV) to quantify the minimum intensity required for unobserved factors. The larger the RV value, the lower the risk that the conclusion is affected by the omitted variables. Using CR and its squared term as the treatment variables, the results show that the RV of the linear term is 12.66%, and the RV of the squared term is 16.43%. This means that to overturn the inverted U-shaped relationship, an unobserved confounding factor needs to explain 12.66% or 16.43% or more of the residual variance of CR (or C*R*^2^) respectively. Referring to the judgment criteria of existing studies, this value falls within the relatively robust to robust range. The boundary analysis further indicates that even if there is an unobserved variable with an intensity 2 to 3 times that of the control variables, the sign direction and significance level of the core coefficient have not undergone a substantive change. In conclusion, the inverted U-shaped conclusion of this study is not sensitive to omitted variable bias and has good robustness.

### 4.4 Moderating effects analysis

This research selected three moderating variables—DIF, RI, and ESO—to identify the moderating mechanisms underlying the inverted U-shaped impact of CR on PH through three climate adaptation pathways (namely financial resilience, economic endowments, and energy infrastructure). Table 5 reports the corresponding results.

**Table 5:** Testing of the moderating effect.

Variables		(1)	(2)	(3)
		PH	PH	PH
CR		0.051* (0.030)	0.069** (0.033)	0.084** (0.034)
C*R*^2^		−0.090** (0.038)	−0.131*** (0.042)	−0.144*** (0.045)
CR × DIF		1.001** (0.434)		
C*R*^2^ × DIF		−1.284** (0.510)		
DIF		0.839*** (0.204)		
CR × RI			0.214* (0.112)	
C*R*^2^ × RI			−0.313** (0.149)	
RI			0.076 (0.066)	
CR × ESO				−0.206*** (0.076)
C*R*^2^ × ESO				0.177** (0.082)
ESO				0.075*** (0.024)
Constant		0.696*** (0.211)	1.035*** (0.222)	1.023*** (0.231)
Control variables		Yes	Yes	Yes
Inverted U-shaped nonlinearity test	Vertex	0.285	0.263	0.290
	Upper limit of the 95% confidence interval	[0.163, 0.407]	[0.161, 0.364]	[0.2008, 0.373]
	Slope at the left endpoint	0.048	0.065	0.076
	Slope at the right endpoint	−0.084	−0.127	−0.126
$\beta_{1}\beta_{4}-\beta_{2}\beta_{3}$		0.026	0.006	−0.015
Changes in the vertex (as M increases)		Move right	Move right	Move left
Change in curvature (as M increases)		steep	steep	gentle
Observations		390	390	390
Within *R*^2^		0.347	0.294	0.295
Province-year fixed		yes	yes	yes

Robust standard errors in parentheses; ***, **, and * indicate that the results are significant at the 1%, 5%, and 10% levels, respectively.

The difference in extreme points between the baseline model and the moderated effect model is due to the re-estimation of the main effect coefficients after the inclusion of interaction terms. This does not represent the direction of the moderated effect; rather, the positive or negative direction of $\left(\beta_{1}\beta_{4}-\beta_{2}\beta_{3}\right)$ truly reflects the direction of the moderated effect.

#### 4.4.1 The moderating effect of DIF

With respect to financial resilience (column (1)), the CR × DIF interaction coefficient is 1.001 and the C*R*^2^ × DIF coefficient is −1.284, both statistically significant. Thus, DIF plays a significant moderating role in the inverted U-shaped CR–PH association, shaping both the curve’s curvature and its vertex. Regarding curvature, *β*₄ is significantly negative (−1.284), implying that the inverted U-shaped relationship grows steeper as DIF becomes more advanced. In other words, in regions with well-developed digital finance, the rate of health adaptation during the low-risk phase is faster, exhibiting an efficiency-enhancing moderating characteristic. Regarding the shift in the vertex, *β*₁β₄ − β₂β₃ = 0.026, indicates that the vertex of the inverted U-curve is displaced rightward by DIF—that is, regions with higher levels of digital finance can withstand greater CR shocks, with digital finance playing a positive role within the carrying capacity of the system. However, this also underscores the need to focus on the resilience of digital infrastructure and energy systems. These findings support Hypothesis 2a.

**Figure 8:**
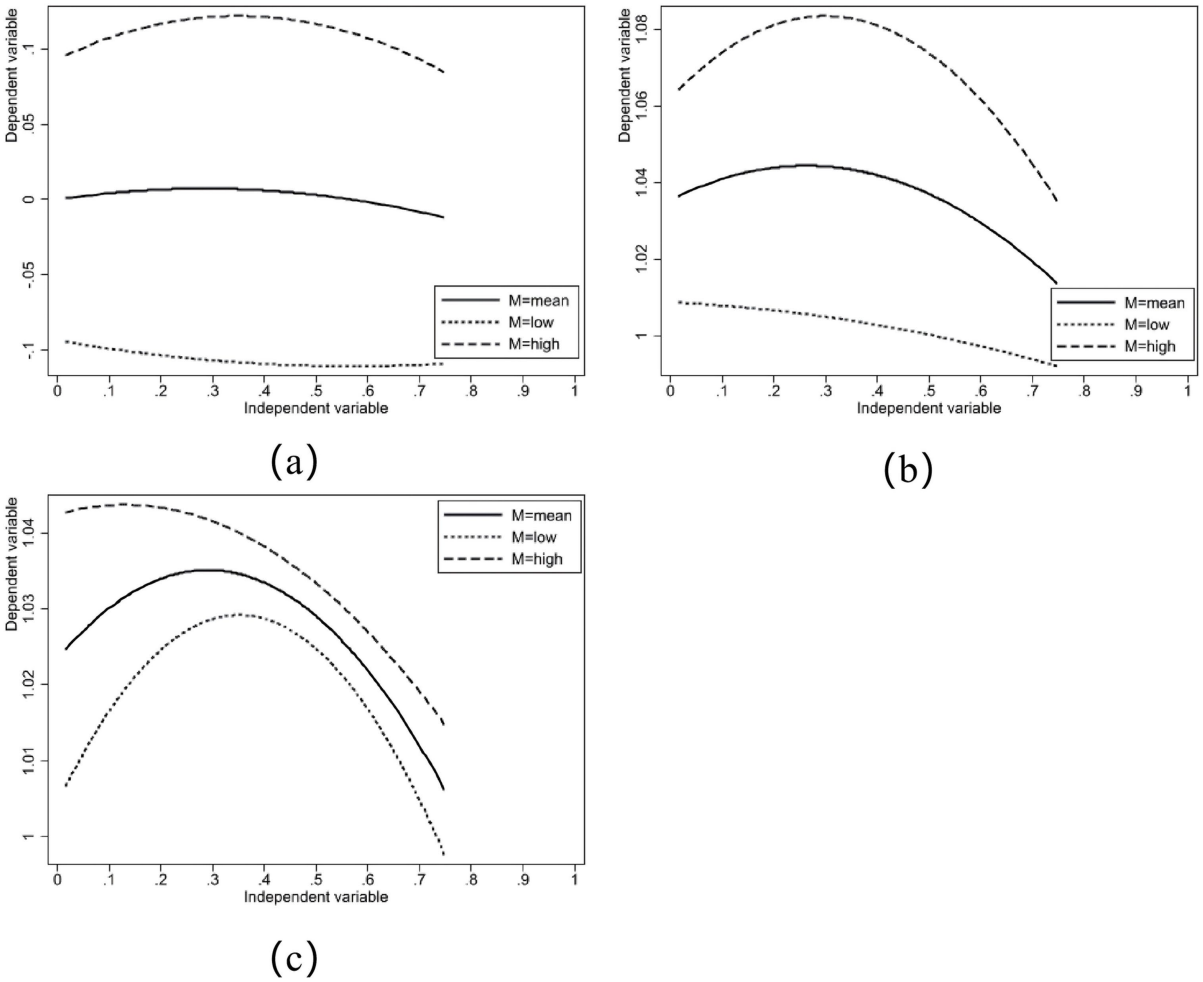
**(a–c)** respectively represent the regulatory effect curves of DIF, RI, and ESO at the low, mean, and high levels. Here, the low level refers to the (mean – std.), and the high level refers to the (mean + std).

Based on the (mean ± standard deviation) method, the (mean - standard deviation) represents a low value, while the (mean + standard deviation) represents a high value. As depicted in Figure 8a, the regulatory effect varies in form depending on the level of DIF. When DIF is at a high level, the relationship is inverted U-shaped, but the curve is steeper, indicating that a high DIF may amplify the marginal effect of climate risks, making the system more sensitive to changes in risks; when DIF is at an average level, the relationship maintains the baseline inverted U-shape; when DIF is at a low level, the relationship is U-shaped, that is, health declines with the increase in risk during the low-risk stage. A reasonable explanation is that in low DIF areas, the lack of digital financial empowerment leads to individuals and communities lacking the most basic preventive tools such as risk warnings, insurance dispersion, and remote medical services. Climate risks are prone to cause significant health losses. The recovery of the curve at the high-risk stage is due to the fact that severe extreme climate events attract government attention and receive assistance, which is delayed but eventually takes effect in low DIF areas.

#### 4.4.2 The moderating effect of RI

Regarding economic resource endowments, as reported in column (2), the CR × RI interaction coefficient is 0.214, reaching only marginal significance, whereas the C*R*^2^ × RI interaction coefficient is −0.313 and attains conventional levels of statistical significance. Thus, RI affects the inverted U-shaped association between CR and PH. Similar to DIF, *β*₄ is significantly −0.313, RI makes the inverted U-shaped curve steeper, but its regulatory intensity is milder. After calculation, the vertex *β*₁β₄ − *β*₂*β*₃ = 0.006. The inverted U-shaped vertex in high-income regions shifts to the right, indicating that under the regulation of income levels, the PH system can withstand a higher CR threshold. It is worth noting that the linear interaction term of RI is only marginally significant, indicating that its positive moderation on the adaptability and resilience of the PH system in the low-risk stage is limited. This might be because the cost of basic adaptation measures (such as fans and basic medical care) is relatively low and affordable for middle- and low-income families, while the high income threshold required for high-level adaptation under extreme risk conditions (such as central air conditioning and high-quality medical care) truly demonstrates its regulatory effect. This discovery reveals the threshold characteristics of the income moderating effect, that is, the marginal protective effect of income levels varies at both ends of the risk distribution.

As shown in the curves of the adjustment effects under different income levels in Figure 8b, the relationship between CR and PH remains an inverted U-shaped trend that first rises and then declines at all low, average, and high values. As the RI increases, the extreme points gradually shift to the right with the increase in RI, and the entire curve becomes sharper and steeper. This evolution pattern further indicates that a high RI delays the turning point of the impact of climate risks on health and simultaneously amplifies the marginal effect of risk changes on health outcomes. In other words, high-income groups can continuously achieve health improvements within a wider risk range, but once the risk exceeds the critical value, the rate of health decline is also faster. At this point, the hypothesis H2b has been verified.

#### 4.4.3 The moderating effect of ESO

At the energy infrastructure level, see the proof in column (3). The coefficient of the ESO × CR is significantly −0.206 at the 1% level, and the coefficient of the ESO × C*R*^2^ is 0.177, with a 5% level significance level. Investigating its influence on the inverted U-shaped curve, unlike DIF and HI, *β*₄ is significantly positive at 0.177 (*p* < 0.05). ESO makes the curvature of the inverted U-shaped curve more gentle. In other words, in regions with a higher share of clean energy generation, PH becomes less sensitive to CR shocks, and a more moderate change trajectory has emerged between CR and health. Inversely, ESO shifts the inverted U-shaped apex to the left (*β*₁*β*₄ − *β*₂*β*₃ = −0.015). This is consistent with the characteristics shown by the ESO modulation curves at different levels (see Figure 8c). When ESO is at a lower level, the curvature of the inverted U-shaped curve is slightly larger, and the extreme point is to the right; as ESO rises to the mean level, the extreme point begins to shift to the left, and the curve becomes more gentle; when ESO is at a higher level, the extreme point shifts further to the left, and the slope of the curve significantly decreases, with both the increase and decrease amplitudes becoming more moderate. This dynamic change means that increasing the proportion of clean energy consumption leads to the positive effect of CR on PH reaching its peak at a lower risk level, then entering a slowly declining range, and overall weakening the marginal impact intensity of CR changes on PH. These findings support Hypothesis H2c.

From the institutional perspective, China’s energy transition exhibits an upwardly-driven characteristic through administrative measures, such as “coal-to-gas” conversion and the “dual carbon” target, which promotes energy cleanliness, enabling PH to benefit even before CR reaches a relatively high level. This policy-driven healthy benefit is released prematurely, which is fundamentally different from the energy transition in Western markets that is led by market forces. Currently, China has established the largest and most complete clean energy industry chain in the world. The initial stage of the ESO often involves the rapid replacement of high-pollution coal power, during which the improvement in health is more significant. When the proportion of clean energy reaches a certain level, the cost of further optimization increases while the increment of health benefits tends to level off, precisely revealing the objective law that energy transition is entering the deep waters of systematic reconfiguration. It is worth noting that recent studies have also found that the accessibility of natural gas pipelines has a U-shaped regulatory effect on the relationship between energy transition and individual welfare (58), which is consistent with the discovery of the leftward shift of the extreme point in this study, indicating that the spatial distribution of energy infrastructure also affects the non-linear characteristics of the climate risk-health relationship. Moreover, in regions where the coal industry has shifted, there is a U-shaped relationship between industrial development and residents’ health (59), and this study found that the ESO shifts the leftward extreme point, jointly revealing that the morphological transformation of the relationship between energy activities and PH is influenced by the specific stage of industrial adjustment and the intensity of policy intervention. In conclusion, in the context of China’s energy transition driven by administrative orders and with a large-scale and extensive scale, the regulatory effect of ESO reflects the institutional advantage of concentrating efforts to achieve major tasks and the objective constraints of marginal returns diminishing over time at each stage.

## 5 Heterogeneity analysis

In addition to the aforementioned moderating mechanisms, the relationship pattern between CR and CR may vary significantly depending on regional characteristics. In fact, environmental regulation, green technological innovation, and green space construction may intervene in the process of CR’s impact on PH through different means such as risk control, technology transfer, and shock absorption. Therefore, this section mainly focuses on the heterogeneous effects generated by three types of regional characteristic variables: the intensity of environmental regulation, green technological innovation, and the level of green space construction. Based on this, we attempt to establish an analytical framework where “institution - technology - ecology” interact with each other, and use this framework to explore the boundary conditions of the inverted U-shaped relationship. Here, this research mainly employs the interaction term model as the main method, and uses mean group regression as a supplementary verification. Among them, group regression uses the sample mean as the grouping criterion, and bootstrap Fisher’s permutation tests based on 500 repeated draws indicate that, across the three heterogeneity analyses, the between-group coefficients for CR and C*R*^2^ are not significantly different (*p* < 0.01). The corresponding outcomes are set out in detail in Tables 6, 7.

**Table 6:** Heterogeneity test: interaction term method.

Variables	(1)	(2)	(3)
	PH	PH	PH
CR	0.107*** (0.037)	−0.093 (0.152)	0.320*** (0.082)
C*R*^2^	−0.213*** (0.052)	0.251 (0.190)	−0.441*** (0.123)
CR × environmental regulation intensity	−1.434 (3.310)		
C*R*^2^ × environmental regulation intensity	15.074** (6.777)		
CR × green technology innovation		0.028 (0.020)	
C*R*^2^ × green technology innovation		−0.061** (0.027)	
CR × green space construction			−0.390*** (0.128)
C*R*^2^ × green space construction			0.509** (0.215)
Constant	1.016*** (0.223)	0.984*** (0.241)	1.058*** (0.236)
Vertex	0.251	0.184	0.363
Control variables	Yes	Yes	Yes
Observations	390	390	390
*R*-squared	0.312	0.295	0.295
Province-year fixed	yes	yes	yes

Robust standard errors in parentheses; ***, **, and * indicate that the results are significant at the 1%, 5%, and 10% levels, respectively.

**Table 7:** Heteroscedasticity test: grouped regression.

Variables	(1)	(2)	(3)	(4)	(5)	(6)
	Low environmental regulation intensity	High environmental regulation intensity	Low green technology innovation	High green technology innovation	Low green space construction	High green space construction
CR	0.150*** (0.053)	0.069 (0.048)	−0.023 (0.030)	0.156** (0.068)	0.117** (0.053)	−0.006 (0.037)
C*R*^2^	−0.235*** (0.077)	−0.109* (0.055)	0.008 (0.036)	−0.219** (0.100)	−0.201*** (0.066)	−0.002 (0.049)
Constant	1.272*** (0.273)	0.679 (0.630)	0.248 (0.297)	1.210*** (0.386)	0.625 (0.589)	0.968*** (0.264)
Control variables	Yes	Yes	Yes	Yes	Yes	Yes
Vertex	0.314	0.317		0.356	0.291	0.370
*p*-value for differences in coefficients between CR groups	0.018		0.000		0.006	
*p*-value for differences in coefficients between C*R*^2^ groups	0.006		0.000		0.002	
Observations	253	136	188	201	190	199
Province-year fixed	yes	yes	yes	yes	yes	yes
Within *R*^2^	0.234	0.396	0.430	0.173	0.462	0.352

Robust standard errors in parentheses; ***, **, and * indicate that the results are significant at the 1%, 5%, and 10% levels, respectively.

When controlling for province and year fixed effects, the low environmental regulation subgroup, the high green technology innovation subgroup, and the high green space construction subgroup had 1 singleton observations, respectively, which were automatically dropped. This does not affect the robustness of the results.

### 5.1 Heterogeneity of environmental regulation intensity

Adopting the measure used by Liu and Xu (60), environmental regulation intensity is defined as the share of industrial pollution treatment investment in industrial output value. Separate regressions were run after dividing the sample into low- and high-environmental regulation intensity groups. The interaction term results [see Table 6 (columns (1))] show that the coefficient of the quadratic interaction term is significantly positive, while the linear interaction term is not significant. This indicates that environmental regulations mainly regulate the curvature of the inverted U-shaped curve rather than its slope. As the regulatory intensity increases, the absolute value of the negative curvature decreases, and the curve becomes more gentle. The grouped regression results indicate that [see Table 7 (columns (1) and (2))], the inverted U-shaped CR–PH relationship is significant in the low environmental regulation subgroup but no longer holds in regions with high environmental regulation intensity, which is due to the decline in statistical power caused by the flattening of the curve. Strict environmental regulation, by limiting pollutant emissions and promoting green transformation of enterprises, not only mitigates climate change at the source and reduces the frequency of extreme weather events but also directly improves the ecological environment in which people live. Overall, environmental regulations effectively buffer the marginal impact of CR on PH, making the health systems in high-regulation areas exhibit greater stability, confirming the positive policy effect of environmental regulations.

### 5.2 Heterogeneity of green technology innovation

From a natural-resource-based perspective, green technological innovation possesses the strategic capabilities for pollution prevention, product management, and promoting sustainable development (61). Given that green patents directly reflect corporate innovation outcomes in environmentally friendly, energy-saving, and clean energy technologies, most studies measure it using green patents data (62). Moreover, compared with patent filings, granted patents are generally considered a better measure of market value and emission reduction potential. Therefore, this paper approximates the level of green technology innovation using the number of granted green patents per 10,000 residents in each province. The results of the interaction term model (column (2) of Table 6) show that the coefficient of the quadratic interaction term is significantly negative (−0.0607, *p* < 0.05), while the coefficient of the linear interaction term is not significant. This indicates that green technological innovation mainly regulates the curvature of the inverted U-shaped curve. As the level of green technological innovation increases, the absolute value of the negative curvature increases, and the curve becomes steeper. The grouped regression results further confirm this finding [columns (3)–(4) of Table 7], the inverted U-shaped CR–PH relationship is significant in the high-innovation group but not obvious in the low-innovation group. This is to say that green technological innovation is an important factor in the inverted U-shaped relationship between climate risks and public health.

In regions with a low level of green technology innovation, there is a lack of key technologies such as clean energy alternatives, pollution control facilities, and climate adaptation technologies. The absence of a refined monitoring and early warning system further disrupts the information chain between risk perception and protective behaviors, making the technology deficiency effect dominant. At this point, the marginal effect of CR on PH is small, and thus it is not statistically significant and shows a flat relationship. When green technology innovation reaches a certain level, the technology empowerment effect begins to emerge. Under a moderate CR, the use of clean energy reduces pollution exposure and improves energy efficiency, green infrastructure mitigates extreme temperatures, and adaptive technologies enhance individual protection capabilities, jointly promoting the improvement of PH (63), forming an ascending segment of the inverted U-shaped curve. However, when CR exceeds the critical value of the technical response capacity, extreme events lead to infrastructure damage, the medical system being under pressure, and residents’ adaptability being exhausted, resulting in negative health marginal benefits and entering the descending segment. The limited technical capabilities and the uncertainty of risk shocks have formed the downward characteristic of the right side of the inverted U-shaped curve.

### 5.3 Heterogeneity of green space construction

The differences in the level of green space construction will alter the buffering capacity of the urban ecosystem against climate risks, which makes green space construction potentially a source of heterogeneity in the relationship between CR and PH. Referring to the measurement method of Wu et al. (64), this paper characterizes the level of green space construction by the green coverage rate of the built-up area. As shown in the interaction term method results in Table 6 (3), the coefficients of the first interaction term and the second interaction term are significantly −0.3897 and +0.5091, respectively, indicating that as the level of green space construction increases, the curve becomes more gentle. The grouped regression results further show (Columns (5)–(6) of Table 7) that the C*R*^2^ coefficient is significantly negative in the low green coverage group, and the inverted U-shaped relationship holds, while it is not the case in the high green space construction group.

In fact, this heterogeneity reflects the buffering effect of green space construction. In areas with a low green coverage rate, green infrastructure is relatively scarce, and the marginal impact of the CR change on PH is more significant. There is a clear inverted U-shaped relationship between the two. In areas with a high green coverage rate, adequate vegetation coverage can effectively regulate local climate, purify the air, and manage rainwater runoff (65, 66). This enables the PH impact of CR to be absorbed and buffered, and thus the marginal effect is weakened accordingly.

## 6 Conclusion and policy implications

### 6.1 Conclusion

Using the panel data of 30 provinces in China (excluding Tibet) from 2011 to 2023, this research investigated the nonlinear impact of CR on PH by constructing a fixed effects model and an UTEST test and identified three moderating mechanisms and heterogeneous effects under the theoretical framework of “finance-economy-energy.” The main conclusions are as follows:

(1) The association between CR and PH follows a pronounced inverted U-shaped curve. After formal verification by UTEST, when the climate risk is below 0.285, the PH system has a strong risk adaptability and can effectively buffer the impact of risks through resource allocation and policy response. Once the risk exceeds this threshold, the adaptability tends to be saturated, and climate risk damage begins to accumulate and take the lead.
(2) DIF and RI have made the inverted U-shaped curve steeper and shifted its peak to the right, presenting an efficiency-enhanced adjustment. In regions with high DIF and high RI, the adaptability response of PH in the low-risk stage is more rapid, and the health system can withstand higher exposure to CR. However, once adaptive capacity becomes overloaded in the high-risk phase, the ensuing health impacts are also more severe. This suggests that while financial resilience and economic resource endowments improve system responsiveness, they may also accelerate the accumulation of health damages during the high-risk phase due to infrastructure dependence and structural vulnerabilities.
(3) ESO flattens the inverted U-shaped curve and shifts its vertex to the left, exhibiting a risk-mitigating moderating pattern. An increased share of clean energy in electricity generation, by reducing air pollutant emissions, improving environmental quality, and enhancing the resilience of the energy system, effectively reduced the marginal impact of CR on PH, enabling health peaks to be reached at lower risk levels and slowing the rate of decline following those peaks.
(4) Fourth, the heterogeneity analysis reveals the boundary conditions of the inverted U-shaped relationship from three dimensions: institutional, technological, and ecological. The results indicate that the intensity of environmental regulations and the development of green spaces exhibit similar buffering effects; at high levels, both cause the inverted U-shaped curve to flatten or even disappear, suggesting that strict environmental oversight and sufficient green coverage can effectively mitigate the health impacts of climate risks. In contrast, green technology innovation exhibits an enabling effect: the inverted U-shaped relationship is significant at high levels of green technology innovation, indicating that green technology innovation is a key factor influencing the nonlinear health effects of climate risks.

### 6.2 Policy recommendations

First, establish a threshold-based early warning mechanism for CR and develop a differentiated, multidimensional climate-health adaptation policy system. Regional governments should implement tiered management according to their local CR levels. For regions that have already exceeded the threshold, policy priorities should focus on risk mitigation, with particular emphasis on strengthening monitoring and early warning systems for extreme weather events, enhancing emergency response capacity, and bolstering healthcare resource reserves. For regions that have not yet exceeded the threshold, efforts should be directed toward raising the system’s carrying capacity by improving social healthcare infrastructure and expanding insurance coverage to enhance health system resilience. Given the significant heterogeneity in health vulnerability to CR across different regions and populations, governments should design a differentiated policy framework that allows local flexibility tailored to specific circumstances. For example, in densely populated areas, policies should focus on preventing the compounding risks of concentrated exposure and infrastructure vulnerability; in medically underserved regions, priority should be given to addressing deficiencies in social healthcare infrastructure. Promote cross-sectoral data sharing and operational coordination among meteorological, health, civil affairs, energy, and other relevant departments, and integrate climate-health risk warnings into the grassroots PH service system to close the “last mile” of policy implementation.

Second, synergistically advance DIF and climate-health adaptation strategies. DIF significantly enhances system risk resilience during the low-risk phase; however, its double-edged sword effect—accelerating health damage during the high-risk phase—cannot be overlooked. Therefore, in regions with better digital finance, concurrent efforts must be made to strengthen the resilience of digital infrastructure, including emergency power supplies, offline payment capabilities, and network redundancy, to mitigate the risk of financial service disruption due to network or power failures during extreme events. At the same time, adaptive financial products—such as climate-indexed insurance and disaster emergency credit—should be promoted, extending the reach of digital services from routine scenarios to extreme event response contexts.

Third, incorporate ESO into the climate-health adaptation policy framework. By improving air quality and enhancing energy system resilience, ESO effectively reduce the sensitivity of CR fluctuations to pH changes. Particularly against the backdrop of China’s “dual carbon” goals, health benefits should be integrated into the cost–benefit assessment of energy transition policies. Priority should be given to deploying distributed energy resources, healthcare facilities, and older population care institutions in areas prone to extreme weather events and regions with poor air quality, thereby forming an “energy–health” resilience coalition.

Finally, strengthen the health synergy effect of green technology innovation, and enhance the climate-health system resilience of environmental regulations and green space construction through two paths: institutional constraints and ecological buffers. Policy formulation should focus on the coordinated cooperation of technology and institutions, and ecological measures. First, jointly promote strict environmental supervision and scientific urban greening. The former strengthens the policy buffering capacity by raising pollution emission standards and health impact assessments, while the latter enhances the ecological service functions of heat environment regulation and air purification by increasing the green coverage rate of built-up areas. In addition, it is essential to increase R&D investment in cleaner production technologies, pollution control technologies, and climate-adaptive technologies. Policy instruments such as tax incentives, green credit, and intellectual property protection should be leveraged to incentivize firms to engage in green technology innovation activities. Meanwhile, a regional coordination mechanism for technology diffusion should be established to facilitate the transfer of proven practices from high-innovation regions to low-innovation regions, thereby narrowing interregional gaps in technological capacity. In conclusion, climate health adaptation policies should shift from a single intervention mindset to a systemic governance mindset. Priority should be given to filling the gaps in regions with weak regulations, lagging innovation, or insufficient greening, in order to comprehensively enhance the overall resilience of the climate-health system.

## Data Availability

Publicly available datasets were analyzed in this study. This data can be found here: https://www.epsnet.com.cn/index.html#/Index.

## Footnotes

1
https://www.tianditu.gov.cn/
